# Degranulation of rat omental mast cells by A_1_ receptor agonists *in vitro*

**DOI:** 10.1155/S096293519600049X

**Published:** 1996-10

**Authors:** A. M. Northover, B. J. Northover

**Affiliations:** Department of Pharmaceutical Sciences School of Applied Sciences De Montfort University Leicester LE1 9BH UK

## Abstract

The haemodynamic effects of adenosine are thought to result in part from a release of mast cell amines via A3 receptor stimulation. To investigate the nature of the receptors involved in adenosine-induced mast cell degranulation in the rat isolated omentum we have used adenosine analogues with varying specificities as activators of the A_1_, A_2_ and A_3_ receptors, and antagonists with differing specificities for A_1_ and A_2_ receptors. Analogues which act predominantly as A_1_ (e.g. N^6^-cyclopentyladenosine) or as mixed A_1_/A_2_ receptor agonists (e.g. adenosine, inosine, 5'-(Nethylcarboxamido) adenosine) caused mast cell degranulation, whereas a predominantly A3 receptor agonist (IB-MECA) was inactive. Pre-treatment of the omentum with the A_1_/A_2_ receptor antagonist 8-phenyltheophylline or with the more specific A_1_ receptor antagonist 8-cyclopentyl-1,3-dipropylxanthine significantly reduced agonist-induced degranulation. Pre-treatment with disodium cromoglycate or with BN52021 also reduced degranulation of mast cells in response to N^6^-cyclopentyladenosine. In the rat isolated omental mast cell we conclude that degranulation is an indirect result of A_1_ receptor stimulation. Platelet-activating factor release appears to mediate at least part of the degranulation.


Mediators of Inflammation, 5, 341–345 (1996)

### Degranulation of rat omental mast cells by $A_1$ receptor agonists *in vitro*

A. M. Northover[CA] and B. J. Northover

Department of Pharmaceutical Sciences, School of Applied Sciences, De Montfort University, Leicester LE1 9BH, UK

[CA]Corresponding Author
Fax: (+44) (0) 116 2577135

**THE haemodynamic effects of adenosine are thought to result in part from a release of mast cell amines via $A_3$ receptor stimulation. To investigate the nature of the receptors involved in adenosine-induced mast cell degranulation in the rat isolated omentum we have used adenosine analogues with varying specificities as activators of the $A_1$, $A_2$ and $A_3$ receptors, and antagonists with differing specificities for $A_1$ and $A_2$ receptors. Analogues which act predominantly as $A_1$ (e.g. $N^6$-cyclopentyladenosine) or as mixed $A_1/A_2$ receptor agonists (e.g. adenosine, inosine, 5′-(N-ethylcarboxamido)adenosine) caused mast cell degranulation, whereas a predominantly $A_3$ receptor agonist (IB-MECA) was inactive. Pre-treatment of the omentum with the $A_1/A_2$ receptor antagonist 8-phenyltheophylline or with the more specific $A_1$ receptor antagonist 8-cyclopentyl-1,3-dipropylxanthine significantly reduced agonist-induced degranulation. Pre-treatment with disodium cromoglycate or with BN52021 also reduced degranulation of mast cells in response to $N^6$-cyclopentyladenosine. In the rat isolated omental mast cell we conclude that degranulation is an indirect result of $A_1$ receptor stimulation. Platelet-activating factor release appears to mediate at least part of the degranulation.**

**Key words:** Adenosine, Adenosine receptors, $N^6$-cyclo-pentyladenosine, 8-cyclopentyl-1,3-dipropylxanthine, 5′(N-ethylcarboxamido)adenosine, IB-MECA, Inosine, Mast cells, 8-phenyltheophylline, Platelet-activating factor

### Introduction

Adenosine has been shown to increase the release of both histamine[1–5] and 5-hydroxytryptamine (5HT)[6] from rat isolated mast cells. This occurs only after such cells have been pre-stimulated with, for example, an antigen[2,3,6] or the ionophore A23187.[1,4,5] Recent evidence suggests that in antigenically stimulated mast cells in culture adenosine increases the release of amines stored in the granules via activation of adenosine type 3 ($A_3$) receptors.[7] Some of the haemodynamic actions of injected adenosine *in vivo* also have been attributed to the release of histamine and/or 5HT from mast cells as a result of activation of $A_3$ receptors.[8–10] The wide heterogeneity among mast cells located in different tissues,[11,12] however, prompted us to explore the extent to which an involvement of $A_3$ receptors is a general phenomenon.

Various methods may be used to study mast cell degranulation. Many workers have used rat isolated peritoneal mast cells,[1–6] but that artificially precludes interactions occurring between normally adjacent cell types. Such interactions form an integral part of inflammation.[12] The rat isolated omentum can be transilluminated and it contains large numbers of connective tissue type mast cells located in the so-called milky spots.[13,14] This makes it possible to observe mast cells in their normal tissue environment, surrounded by various other cell types. Mast cells normally stain metachromatically when they are exposed to toluidine blue,[15] but lose this property after degranulation.[14] In the present work we have investigated the mast cell degranulating influence of applied adenosine and some of its synthetic structural analogues in the rat omentum. The nature of the receptors that are involved in these responses has been explored with the aid of selective adenosine receptor antagonists. We have also investigated whether or not pre-treatment of the omentum with disodium cromoglycate (DSCG) can reduce

© 1996 Rapid Science Publishers the degranulating effect of a specific adenosine type 1 ($A_1$) receptor agonist. In addition, we have investigated the possibility that adenosine receptor agonists indirectly degranulate the mast cells in this preparation by first releasing platelet-activating factor (PAF) or a cyclo-oxygenase product from within the omentum. We have shown previously that both of these types of agent occur endogenously and can degranulate mast cells in the rat omentum *in vitro*.[14]

### Methods

Female rats weighing about 250 g were killed by inhalation of chloroform vapour. The lesser omentum was removed from the abdomen and divided into five pieces of approximately equal size. Each piece was spread out gently on a microscope slide and flooded with normal saline (NS) or with NS plus a putative antagonist, and then incubated in a moist atmosphere at 37°C, as described previously.[14] After 5 min of incubation, the bathing fluid was drained away and replaced with fresh NS, or with NS plus agonist, or with NS plus antagonist plus agonist, or with NS plus antagonist. Incubation continued for a further 15 min. The specimens were then drained, washed with distilled water, and stained for 8 min with a solution of toluidine blue (0.5%) in McIlvaine's buffer (pH 4). After rinsing again with distilled water, the specimens were viewed under a microscope at × 100 magnification. The number of metachromatically (pink/magenta)-stained mast cells in each omental milky spot was counted. The average count for all the milky spots in each piece of omentum was used to calculate the mean ± SEM for each treatment group. Omental pieces from a minimum of three rats were used for each treatment group. The number of milky spots examined varied from 15 to 40 per piece of omentum.

Compounds tested as possible agonists were adenosine itself; inosine, which binds weakly to $A_1$, $A_2$ and $A_3$ receptors;[16] 5′-(*N*-ethylcarboxamido) adenosine (NECA), a mixed $A_1/A_2$ receptor agonist;[16,17] 1-deoxy-1-[6-[[(3-iodophenyl)methyl]amino]-9H-purin-9-yl]-*N*-methyl-β-D-ribofuranuronamide (IB-MECA), which is thought to act predominantly via $A_3$ receptors;[16] and $N^6$-cyclopentyladenosine (CPA), which acts primarily via $A_1$ receptors.[17] Compounds tested as putative antagonists were 8-phenyltheophylline (8PT), a mixed $A_1/A_2$ receptor antagonist;[18] 8-cyclopentyl-1,3 dipropylxanthine (DPCPX), which is considerably more potent as an $A_1$ receptor antagonist than as an $A_2$ antagonist;[19] DSCG, a peritoneal mast cell stabilizer;[20,21] BN52021, a PAF-receptor antagonist;[22] and indomethacin, a cyclo-oxygenase inhibitor.[23]

### Chemicals

Adenosine, inosine, NECA, CPA, 8PT, DPCPX and DSCG were obtained from Sigma Chemical Co. Ltd (Poole, UK); IB-MECA from RBI (Natick, MA, USA); indomethacin from Merck, Sharpe & Dohme Ltd (Hoddesdon, UK); and toluidine blue (batch 9244890D) from BDH (Poole, UK). BN52021 was a gift from Dr P. Braquet, Institut Henri Beaufor (Le Plessis- Robinson, France).

NECA, CPA, 8PT, DPCPX, IB-MECA and BN52021 were each initially dissolved in dimethylsulphoxide, the final concentration of this solvent to which the omentum was subsequently exposed being $< 0.5\%$. Adenosine, inosine, DSCG and indomethacin were used as aqueous solutions, indomethacin being dissolved with the aid of a little $Na_2CO_3$.

### Statistics

Bonferroni's test was used for comparing several treatment groups with one control group.[24]

### Results

Adenosine (1 μM), inosine (100 μM), NECA (0.1 μM) and CPA (0.1 μM) were each found to cause significant degranulation of the mast cells in milky spots of the rat isolated omentum (Fig. 1a,b). Ten-fold lower concentrations of each of these compounds, however, failed to exert a significant degranulating effect (Fig. 1a,b). Ten-fold higher concentrations of adenosine (10 μM) and of NECA (1 μM) were also without a significant degranulating effect (Fig. 1a,b), whereas a ten-fold increase in the concentration of CPA (to 1 μM) caused almost the same amount of degranulation as the lower concentration of 0.1 μM (Fig. 1b). IB-MECA was ineffective in the present experiments in a wide concentration range of 0.1–100 μM (Fig. 1a).

Pre-treatment of the omentum with 8PT (1 μM) significantly reduced the degranulating effects of adenosine (1 μM), inosine (100 μM) and NECA (0.1 μM) (Fig. 1a,b). Pretreatment of the omentum with DPCPX (0.01 μM) significantly reduced the responses to both NECA (0.1 μM) and CPA (0.1 μM) (Fig. 1b). It is important to note that at these concentrations, neither 8PT nor DPCPX alone produced a significant effect on degranulation (mast cell counts $15.3 \pm 0.8$, $17.3 \pm 0.7$ respectively). A ten-fold lower concentration of DPCPX (0.001 μM) failed to reduce the effect of CPA

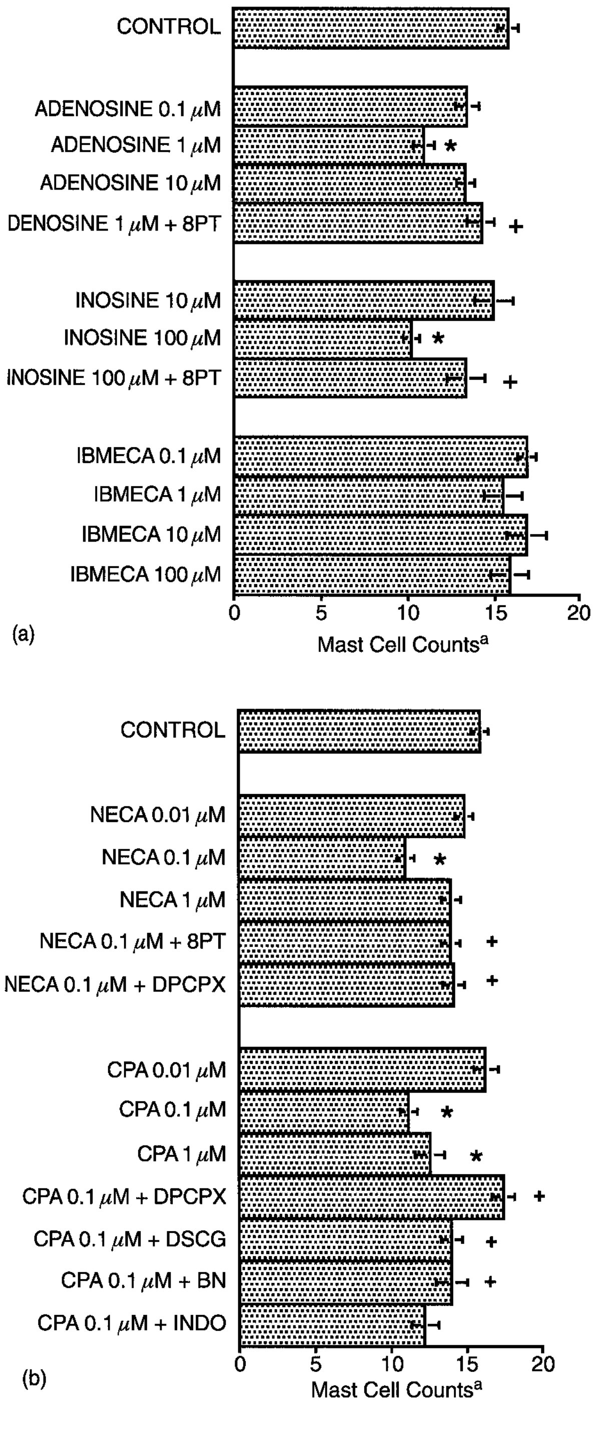

FIG. 1. Effects of various adenosine receptor agonists on the degranulation of rat omental mast cells. [a]Mast cell counts are the mean numbers ± SEM of metachromatically stained mast cells per omental milky spot. *lower than control value ($p < 0.05$, Bonferroni's test); +higher than the value for the relevant agonist alone ($p < 0.05$, Bonferroni's test) after treatment with 8PT (1 μM), DPCPX (0.01 μM) DSCG (100 μM) or BN52021 (10 μM). Abbreviations not used in the text: BN, BN52021; INDO, indomethacin (1 μM).

significantly (mast cell count 11.9 ± 0.8). A ten-fold higher concentration of 8PT (10 μM) actually had a degranulating effect of its own (mast cell count 12.7 ± 0.7). A ten-fold higher concentration of DPCPX (0.1 μM) had no greater effect than 0.01 μM (mast cell count 17.5 ± 1.6).

Pretreatment of the omentum with DSCG (100 μM) or with BN52021 (10 μM) significantly antagonized the effects of added CPA (0.1 μM), whereas indomethacin (1 μM) was inactive under similar conditions (Fig. 1b). DSCG (100 μM) alone had no significant effect on degranulation (mast cell count 16.9 ± 0.7), BN52021 and indomethacin alone having been shown previously to have no degranulating effect at these concentrations.[14]

### Discussion

The present results show that adenosine, inosine, NECA and CPA each degranulate rat omental mast cells *in vitro*. Adenosine,[18] inosine[16] and NECA[16,17] have each been reported to stimulate both the $A_1$ and $A_2$ types of receptor, with NECA being more potent than adenosine in this respect,[17] but with adenosine in turn being more potent than inosine.[16,25] The degranulating effect of each of these agonists in the present experiments was counteracted by pretreating the omentum with 8PT, a mixed $A_1$/$A_2$ receptor antagonist[18] (Fig. 1a,b). In contrast, IB-MECA, which is a much more powerful activator of $A_3$ receptors than of $A_1$ or $A_2$ receptors,[16] unexpectedly showed no effect, even when tested at a concentration of 100 μM (Fig. 1a), suggesting that $A_3$ receptor stimulation was not involved. This is in marked contrast to the observation that $A_3$ activation can cause murine mast cells to degranulate *in vivo*.[9] Moreover, the application of CPA, a relatively specific $A_1$ receptor agonist,[17] caused a significant degree of mast cell degranulation in the present experiments (Fig. 1b). It is important to note also that pretreatment with DPCPX, a relatively specific $A_1$ receptor antagonist,[19] significantly reduced the degranulating effects of both CPA and NECA (Fig. 1b). These results, therefore, strongly suggest that involvement of $A_1$ receptors predominated in the present situation. $A_2$ receptor activation, on the other hand, is unlikely to have been important. A raised level of cyclic AMP, which one might expect to result from any $A_2$ receptor stimulation,[18] does not cause a release of amines from mast cells.[2–4] This also accords with the fact that any $A_2$-stimulating action of NECA which might have been unmasked after blocking its $A_1$ receptor-mediated effect with DPCPX, actually produced no observable effect on degranulation in the present experiments (Fig. 1b). It is worth noting here that other workers have found that 10 μM

adenosine was less active than 1 μM adenosine in reducing vascular luminal diameter, which is another phenomenon thought to be due to mast cell degranulation.[25] The affinity constants at adenosine-sensitive binding sites shown by both adenosine and NECA are higher at $A_2$ than at $A_1$ receptors.[18] The lesser degranulating effect of the higher concentrations (10 μM and 1 μM respectively) of these two compounds may have been due to partial mast cell stabilization exerted via a stimulation of $A_2$ (adenylate cyclase stimulating) receptors that appeared only at these higher concentrations. The degranulating effects exerted via $A_1$ (adenylate cyclase inhibitory) receptors, on the other hand, may already have become maximal at the lower concentration tested (1 μM and 0.1 μM respectively). A similarity in the degranulating effect shown by CPA at 0.1 μM and 1 μM (Fig. 1b) would thus be consistent with its very weak $A_2$ receptor agonist activity.[17]

Those $A_1$ receptors which seem to be responsible for the present observations may reside in the mast cell membranes or they may be located in other cells within the omentum. Activation of the $A_1$ receptors in various types of cell can lead to either an increase or a decrease in the coupling of surface receptors to their G proteins.[26] Hence, mast cell degranulation that was observed here may have been produced indirectly, rather than as a direct result of adenosine receptor stimulation of the mast cell itself. Other workers have put forward a similar proposition.[10] In support of this hypothesis, it was found that pretreatment of the omentum with BN52021, a PAF-receptor antagonist,[22] significantly reduced the degranulating effects of added CPA, although indomethacin, a cyclo-oxygenase inhibitor,[23] did not (Fig. 1b). Under the present experimental conditions, therefore, a release of PAF may mediate part of the degranulation that was seen. This would correlate well with our earlier observation using this preparation, that PAF, and possibly thromboxane $A_2$, appeared to be involved as mediators of the mast cell degranulation that was induced by the NO-synthase inhibitor *N*-nitro-L-arginine methyl ester.[14] However, in addition to histamine, 5-hydroxytryptamine and PAF, various pro-inflammatory cytokines are known to be released from stimulated mast cells.[11,12] We have no evidence to implicate or rule out an involvement of cytokines or other such substances here. It is possible that protection against the degranulating effect of CPA that was shown by DSCG (Fig. 1b) may have resulted from reduced release of one of these other substances,[27] but equally it may have been due to an adenosine receptor blocking action of the type described by earlier workers.[28]

Finally, it must be noted that mast cells vary considerably in their responsiveness to stimulation by different agents.[11,12] One cannot extrapolate with any degree of certainty, therefore, between different organs or species. Mast cells in the rat omentum may turn out to be exceptional. They certainly differ from those in rat skin[29] in being refractory to degranulation with a potent $A_3$ receptor-specific agonist such as IB-MECA, but in being sensitive to a potent $A_1$-receptor selective agonist such as CPA. Further work is needed to explore the pharmacological responsiveness of mast cells located in different rat tissues.

### References

1. Marquardt DL, Parker CW, Sullivan TJ. Potentiation of mast cell mediator release by adenosine. *J Immunol* 1978; **120**: 871–878.
2. Church MK, Hughes PJ. Adenosine potentiates immunological histamine release from rat mast cells by a novel cyclic AMP-independent cell-surface action. *Br J Pharmacol* 1985; **85**: 3–5.
3. Leoutsakos A, Pearce FL. The effect of adenosine and its analogues on cyclic AMP changes and histamine secretion from rat peritoneal mast cells stimulated by various ligands. *Biochem Pharmacol* 1986; **35**: 1373–1379.
4. Lohse MJ, Maurer K, Gensheimer H-P, Schwabe U. Dual actions of adenosine on rat peritoneal mast cells. *Naunyn-Schmiedeberg's Arch Pharmacol* 1987; **335**: 555–560.
5. Lohse MJ, Maurer K, Klotz K-N, Schwabe U. Synergistic effects of calcium-mobilizing agents and adenosine on histamine release from rat peritoneal mast cells. *Br J Pharmacol* 1989; **98**: 1392–1398.
6. Church MK, Hughes PJ, Vardey CJ. Studies on the receptor mediating cyclic AMP-independent enhancement by adenosine of IgE-dependent mediator release from rat mast cells. *Br J Pharmacol* 1986; **87**: 233–242.
7. Ramkumar V, Stiles GL, Beaven MA, Ali H. The $A_3$ adenosine receptor is the unique adenosine receptor which facilitates release of allergic mediators in mast cells. *J Biol Chem* 1993; **268**: 16887–16890.
8. Hannon JP, Pfannkuche HJ, Fozard JR. A role for mast cells in adenosine $A_3$ receptor-mediated hypotension in the rat. *Br J Pharmacol* 1995; **115**: 945–952.
9. Fozard JR, Pfannkuche H-J, Schuurman H-J. Mast cell degranulation following adenosine $A_3$ receptor activation in rats. *Eur J Pharmacol* 1996; **298**: 293–297.
10. Shepherd RK, Linden J, Duling BR. Adenosine-induced vasoconstriction *in vivo*. Role of the mast cell and $A_3$ adenosine receptor. *Circ Res* 1996; **78**: 627–634.
11. Foreman JC. Mast cells and basophil leucocytes. In: Dale MM, Foreman JC, Fan T-PD, eds. *Textbook of Immunopharmacology*. London: Blackwell Scientific, 1994; 21–34.
12. McNeil HP. The mast cell and inflammation. *Aust NZ J Med* 1996; **26**: 216–225.
13. TB Johnston, Whillis J, eds. *Grays Anatomy Descriptive and Applied*. Thirtieth edition. London: Longmans, Green, 1949; 1356–1357.
14. Northover AM, Northover BJ. Inhibition of NO-synthase and degranulation of rat isolated mast cells *in vitro*. *Mediators of Inflammation* 1996; **5**: in press.
15. Enerbäck L. Mast cells in rat gastrointestinal mucosa. 2. Dye-binding and metachromatic properties. *Acta Path et Microbiol Scandinav* 1966; **66**: 303–312.
16. Gallo-Rodriguez C, Ji X-D, Melman N, Siegman BD, Sanders LH, Orlina J, Fisher B, Pu Q, Olah ME, van Galen PJM, Stiles GL, Jacobson KA. Structure-activity relationships of $N^6$-benzyladenosine-5′-uronamides as $A_3$-selective adenosine agonists. *J Med Chem* 1994; **37**: 636–646.
17. Hamilton HW, Taylor MD, Steffen RP, Haleen SJ, Bruns RF. Correlation of adenosine receptor affinities and cardiovascular activity. *Life Sci* 1987; **41**: 2295–2302.
18. Daly JW. Adenosine receptors: targets for future drugs. *J Med Chem* 1982; **25**: 197–207.
19. Bruns RF, Fergus JH, Badger EW, Bristol JA, Santay LA, Hartman JD, Hays SJ, Huang CC. Binding of the $A_1$-selective adenosine antagonist 8-cyclopentyl-1,3-dipropylxanthine to rat brain membranes. *Naunyn-Schmiedeberg's Arch Pharmacol* 1987; **335**: 59–63.
20. Orr TSC, Hall DE, Gwilliam JM, Cox JSG. The effect of disodium cromoglycate on the release of histamine and degranulation of mast cells induced by compound 48/80. *Life Sci* 1971; **10**: 805–812.
21. Kusner EJ, Dubnick B, Herzig DJ. The inhibition by disodium cromoglycate *in vitro* of anaphylactically induced histamine release from rat peritoneal mast cells. *J Pharmacol Exp Ther* 1973; **184**: 41–46.
22. Braquet P, Godfroid JJ. PAF-acether specific binding sites: 2. Design of specific antagonists. *Trends Pharmacol Sci* 1986; **7**: 397–403.
23. Vane JR. Inhibition of prostaglandin synthesis as a mechanism of action of aspirin-like drugs. *Nature (Lond)* 1971; **231**: 232–239.
24. Wallenstein S, Zucker CL, Fleiss JL. Some statistical methods useful in circulation research. *Circ Res* 1980; **47**: 1–9.
25. Doyle MP, Linden J, Duling BR. Nucleoside-induced arteriolar constriction: a mast cell-dependent response. *Am J Physiol* 1994; **266**: H2042–H2050.
26. Linden J. Structure and function of $A_1$ adenosine receptors. *FASEB J* 1991; **5**: 2668–2676.
27. Richards IM, Dixon M, Jackson DM, Vendy K. Alternative modes of action of sodium cromoglycate. *Agents Actions* 1986; **18**: 294–300.
28. Marquadt DL, Wasserman SI. [$^3$H]Adenosine binding to rat mast cells—pharmacologic and functional characterization. *Agents Actions* 1985; **16**: 454–461.
29. Jones CA, Reeves JJ, Sheehan MJ, Whelan CJ. Adenosine $A_3$ receptors mediate a mast cell-dependent plasma protin extravasation in rat skin. *Br J Pharmacol* 1996; **118**: 152P.

**Received 16 July 1996;**
**accepted in revised form 27 August 1996**
